# Factors Associated With the Experience of Cognitive Training Apps for the Prevention of Dementia: Cross-sectional Study Using an Extended Health Belief Model

**DOI:** 10.2196/31664

**Published:** 2022-01-14

**Authors:** Jaegyeong Lee, Jung Min Lim

**Affiliations:** 1 College of Nursing Seoul National University Seoul Republic of Korea; 2 Department of Pharmacology Yonsei University College of Medicine Seoul Republic of Korea

**Keywords:** cognitive training apps, dementia knowledge, health belief model, middle-aged, logistic regression analysis, dementia, Alzheimer disease, cognition, mobile apps, health apps

## Abstract

**Background:**

The prevalence and economic burden of dementia are increasing dramatically. Using information communication technology to improve cognitive functions is proven to be effective and holds the potential to serve as a new and efficient method for the prevention of dementia.

**Objective:**

The aim of this study was to identify factors associated with the experience of mobile apps for cognitive training in middle-aged adults. We evaluated the relationships between the experience of cognitive training apps and structural variables using an extended health belief model.

**Methods:**

An online survey was conducted on South Korean participants aged 40 to 64 years (N=320). General characteristics and dementia knowledge were measured along with the health belief model constructs. Statistical analysis and logistic regression analysis were performed.

**Results:**

Higher dementia knowledge (odds ratio [OR] 1.164, *P*=.02), higher perceived benefit (OR 1.373, *P*<.001), female gender (OR 0.499, *P*=.04), and family history of dementia (OR 1.933, *P*=.04) were significantly associated with the experience of cognitive training apps for the prevention of dementia.

**Conclusions:**

This study may serve as a theoretical basis for the development of intervention strategies to increase the use of cognitive training apps for the prevention of dementia.

## Introduction

### Background

Population aging is a worldwide phenomenon, and the proportion of the elderly is increasing dramatically in developed countries. Along with this trend, the prevalence of dementia, which mainly affects older people, is also growing substantially. Currently, 6.2 million (11.3%) Americans aged 65 years or older have Alzheimer disease (AD), which is expected to grow more than twice by 2050 [1]. Similarly, 0.75 million (10.2%) South Koreans aged 65 or older are living with dementia, which is estimated to reach 3 million (16.1%) by 2050 [2]. The cost of health care for dementia patients is also becoming a huge burden, annually spending US $355 billion in the United States [1] and US $1 billion in South Korea [2].

Although AD commonly affects the elderly, the first patient reported was a middle-aged person [3]. Early-onset AD, which refers to a diagnosis of AD before the age of 65 years, accounts for about 5% of all AD [4]. The main symptoms may seem similar, although the diagnosis is greatly delayed and shows a more aggressive course of disease [5]. General management strategies for early-onset AD are similar to senile AD, but targeted cognitive therapies and age-appropriate psychosocial support are critical [5]. Awareness of the disease entity is relatively low, and most studies on AD are focused on adults over 65 years of age, leaving out the early onset population. The middle-aged are a unique age group. There are many middle-aged people who, on the one hand, experience with parents or relatives suffering from dementia; on the other hand, many of them are susceptible to early-onset AD. Therefore, this group deserves more attention.

Numerous preventive measures may help manage the growing burden of AD. Meta-analysis suggested that if some modifiable risk factors for AD were reduced by 10-20% per decade, the prevalence of dementia in 2050 could be decreased by 8-15% [6]. Moreover, engaging in mentally stimulating activities can greatly decrease the risk of developing AD [7], and low education attainment is known as the largest modifiable risk factor for AD development [8]. Therefore, early intervention of risk factors and active prevention measures may greatly decrease the socioeconomic burden of dementia.

Behavioral changes leading to the prevention of dementia are greatly influenced by personal beliefs that dementia can be prevented or by personal experiences with patients with dementia [9]. These perceptions about dementia can also be negatively affected by inaccurate knowledge about dementia. If someone’s level of awareness for the causes and symptoms of dementia is low, they are less likely to participate in preventive activities for dementia, which may lead to delayed diagnosis and treatment [10,11]. Therefore, knowledge and perceptions about dementia is very important for the proper management of the disease [12]. However, according to a systematic review on the knowledge about dementia in the general population, 19 out of 40 reports showed that the level of dementia knowledge was low or very limited [13]. Another study found that the middle-aged population showed low levels of knowledge regarding dementia risk factors [14,15]. Therefore, the middle-aged may perceive dementia as a nonpreventable disease and may be less likely to take preventive measures.

Today, various mobile apps are being developed and utilized for the early detection or prevention of health conditions. Apps developed for dementia prevention mainly focus on cognitive training or stimulation, which may improve cognitive functions such as memory, concentration, or visuospatial coordination [16-20]. Many studies have shown that using these apps can improve memory and enhance quality of life, both in healthy adults and individuals with mild cognitive impairment [20-24].

Despite the reported usefulness of this technology, factors associated with the use of cognitive training apps for dementia prevention have not been reported in the literature. The use of cognitive training apps for dementia prevention is a combination of health-related behavior and the acceptance of technology. The health belief model (HBM) is widely used to predict the determinants related to health-related behaviors, which evaluates constructs including perceived benefits, perceived barriers, perceived susceptibility, and perceived severity to understand the likelihood of behavior [25]. The technology acceptance model is used to evaluate the causes that affect people to accept or reject technology, which measures perceived ease of use and perceived usefulness to explain usage intentions and behavior [26,27]. As each model has its limitations to explain the use of health-related apps, many recent studies have attempted to extend or combine models for better explanations of the factors that affect the acceptance of mobile health care apps [28-31].

### Objective

The aim of this study was to investigate the association of structural variables and perceived health belief constructs with the experience of cognitive training apps in middle-aged adults, based on the HBM. Perceived benefits and barriers were measured for the use of cognitive training apps, and perceived susceptibility and severity was measured for dementia. Additionally, we included dementia knowledge as an additional variable to increase the explanatory power of our model.

## Methods

### Recruitment

Data were collected from middle-aged adults in South Korea aged 40 to 64 years between February 4 and February 8, 2021. The participants were recruited online by a professional agency (Macromill Embrain Co), where about 1.3 million participants from the general population are maintained by a demographic distribution based on census data from the National Statistical Office. First, a weblink or notice was sent to the participants via email or mobile app, whereby all participants were informed about the purpose of the study via online documentation on the starting page. The information was available for download if needed. The participants voluntarily moved onto the survey by clicking the start button, which was considered as an informed consent. Next, the participants were asked to respond to an online questionnaire. The full survey is provided in Multimedia Appendices 1 and 2. One of the unique features of an online survey is that the survey does not progress to the next question if an answer is omitted or inaccurate, which prevents incomplete or inaccurate data. The participants were free to drop out at any point of the survey if they wanted to. All personal identifying information was removed from the collected data. This study was approved by the institutional review board of Seoul National University (2102/002-002).

A total of 547 participants initially accessed the survey; 2 people did not satisfy the age criteria, and 39 dropped out before completion. Of the remaining 506 participants, 362 were selected using the proportionate quota sampling method for age and gender, due to oversampling. Participants in their 40s, 50s, and 60s were included at a 2:2:1 ratio, and men and women were selected at a 1:1 ratio, respectively. Of the selected 362 people, 42 were excluded due to poor data quality, which included extremely short response times or giving same answers for all items. In conclusion, 320 participants were included in the final analysis.

### Measures

The participants rated each of the following items on a 5-point Likert scale (1=strongly disagree to 5=strongly agree), unless stated otherwise.

#### General Characteristics

Age, gender, education level, marital status, chronic diseases, family history of dementia, and experience of using cognitive training apps were assessed with standard survey items.

#### Dementia Knowledge

Dementia knowledge was measured with a dementia awareness scale developed for a national survey on the prevalence of dementia in South Korea [2], which evaluates the individual’s knowledge about various aspects of dementia. The scale consists of 15 items which are answered “yes,” “no,” or “don’t know” (to account for false positives), and the number of correct answers is summed up to a final score.

#### Perceived Benefit and Barrier of Using Cognitive Training Apps

The perceived benefit of using apps for cognitive training was measured with 4 items adapted from Venkatesh and Davis [27]. The questions were originally used to measure the perceived usefulness of accepting technology, which were modified for using apps and adapted in Korean for this study. The perceived barrier of using apps for cognitive training was measured with a modified set of 5 items described previously [32]. The items were originally used to measure the perceived barriers of using mobile health apps, which were modified for cognitive training apps in this study.

#### Perceived Susceptibility and Severity of Dementia

The perceived susceptibility of dementia and severity of dementia was measured with 4 items each, derived from the intention-to-screen questionnaire originally described by Galvin et al [33] and adapted in Korean by Yoo and Kim [34].

### Statistical Analysis

Data were analyzed with SPSS, version 22.0 (IBM Corp). The differences between the groups were analyzed using the Student *t* test or one-way analysis of variance (ANOVA), and *P*<.05 was considered statistically significant. We performed logistic regression analysis to examine the relationships between the measured variables and the experience of cognitive training apps.

## Results

### General Characteristics

The general characteristics of the study sample are shown in Table 1. Of the 320 respondents, 82 (25.6%) had experience with cognitive training apps, while 238 (74.4%) did not. We compared the general characteristics between these 2 groups. Within the study sample, 62.2% (n=51) of participants from the experienced group were female, compared to 47.1% (n=112) for the nonexperienced group (*X*^2^=5.591, *P*=.02). Participants with a family history of dementia accounted for 29.3% (n=24) in the experienced group, which was significantly higher compared with 16.8% (n=40) for the nonexperienced group (*X*^2^=5.919, *P*=.02).

**Table 1:** Comparison of general characteristics according to experience of cognitive training apps (N=320).

Variables		All participants		Experience of cognitive training apps					
		(N=320)		Yes (n=82)		No (n=238)	*X* ^2^		*P* value
**Age range (years), n (%)**							2.850		.24
	40-49	124 (38.8)	26 (31.7)		98 (41.2)				
	50-59	129 (40.3)	39 (47.6)		90 (37.8)				
	60-64	67 (20.9)	17 (20.7)		50 (21.0)				
**Gender, n (%)**							5.591		.02
	Male	157 (49.1)	31 (37.8)		126 (52.9)				
	Female	163 (50.9)	51 (62.2)		112 (47.1)				
**Education level, n (%)**							2.506		.29
	High school	74 (23.1)	23 (28.0)		51 (21.4)				
	College	211 (65.9)	53 (64.6)		158 (66.4)				
	Graduate school	35 (10.9)	6 (7.3)		29 (12.2)				
**Marital status, n (%)**							0.366		.83
	Single	40 (12.5)	9 (11.0)		31 (13.0)				
	Married	256 (80.0)	65 (80.5)		190 (79.8)				
	Other	24 (7.5)	7 (8.5)		17 (7.1)				
**Chronic diseases, n (%)**							0.381		.54
	Yes	112 (35.0)	31 (37.8)		81 (34.0)				
	No	208 (65.0)	51 (62.2)		157 (66.0)				
**Family history of dementia, n (%)**							5.919		.02
	Yes	64 (20.0)	24 (29.3)		40 (16.8)				
	No or other	256 (80.0)	58 (70.7)		198 (83.2)				

### Descriptive Statistics of Study Variables

Descriptive statistics of the measured study variables according to experience of cognitive training apps are shown in Table 2. The participants with experience of cognitive training apps showed higher levels of dementia knowledge (*P*<.001) and perceived benefit of using cognitive training apps (*P*<.001), compared with nonexperienced individuals. The perceived barrier of using cognitive training apps was lower (*P*=.02) in the experienced group. Both perceived susceptibility and severity of dementia did not show significant differences between the groups.

**Table 2:** Comparison of measured variables according to experience of cognitive training apps (N=320).

Variables	All participants	Experience of cognitive training apps			
	(N=320)	Yes (n=82)	No (n=238)	*t* value	*P* value
Dementia knowledge, mean (SD)	9.05 (2.28)	9.87 (2.42)	8.77 (2.17)	3.817	<.001
Perceived benefit of using apps, mean (SD)	14.92 (2.47)	16.16 (2.12)	14.50 (2.45)	5.875	<.001
Perceived barrier of using apps, mean (SD)	13.44 (3.08)	12.76 (3.27)	13.68 (2.98)	-2.354	.02
Perceived susceptibility of dementia, mean (SD)	11.45 (2.83)	11.04 (3.05)	11.59 (2.75)	-1.524	.13
Perceived severity of dementia, mean (SD)	14.51 (2.40)	14.62 (2.54)	14.47 (2.35)	.506	.61

### Factors Associated With the Experience of Cognitive Training Apps

Based on the statistical analyses above, we included 2 general characteristics (gender and family history of dementia) and 3 measured variables (dementia knowledge, perceived benefit, and perceived barrier) as possible predicting factors of the experience of cognitive training apps for logistic regression analysis (Table 3). The results revealed that higher dementia knowledge (odds ratio [OR] 1.164, *P*=.02), higher perceived benefit (OR 1.373, *P*<.001), female gender (OR 0.548, *P*=.04), and family history of dementia (OR 1.933, *P*=.04) showed positive relationships with experience of cognitive training apps for the prevention of dementia.

**Table 3:** Predicting factors of experience of cognitive training apps.

Variables	B^a^	SE	OR^b^	95% CI	*P* value
Dementia knowledge	0.152	0.067	1.164	1.021-1.328	.02
Perceived benefit of using apps	0.317	0.072	1.373	1.192-1.581	<.001
Perceived barrier of using apps	-0.033	0.047	0.499	0.480-0.967	.48
Gender (male=1)	-0.601	0.285	.548	0.314-0.958	.04
Family history of dementia (yes=1)	0.659	0.327	1.933	1.018-3.669	.04
Constant	-8.314	1.882	0.000	—^c^	—

^a^B: unstandardized regression weight.

^b^OR: odds ratio.

^c^Not applicable.

## Discussion

### Principal Findings

This study investigated the factors associated with the actual use of cognitive training apps for the prevention of dementia in middle-aged adults. Among the 320 participants, only 82 (25.6%) reported to have experience with cognitive training apps, which is still quite low, considering the widespread distribution and frequent usage of smartphones and related apps in South Korea. As the amount of evidence on the efficacy of cognitive training apps for improving cognitive functions or preventing dementia is increasing [21-23], facilitating the use of mobile apps for dementia care could be an easy solution with multiple positive effects. Conventional offline dementia prevention programs require physical space and human resources, and physical disabilities of an individual may limit access to the program [35]. Innovative programs that utilize mobile apps can overcome these hurdles, which may also lead to continued use. In addition, gamified cognitive training apps are fun and motivational, cost very little, and can be performed in a comfortable environment at a convenient time [36], which could greatly increase adherence. However, one must note that not all cognitive training apps are based on scientific evidence, and therefore these ideas should not be generalized. Further studies investigating the effects of specific cognitive training apps and the types of cognitive training included are needed, which would shed light on how to select a cognitive training app for the prevention of dementia.

Our results showed that the perceived benefit of using cognitive training apps was positively associated with the use of cognitive training apps, but other HBM constructs did not. Previous studies also suggest that perceived benefit is a consistent predictor of health-related behavior, while perceived susceptibility and severity often failed to explain health-related behavior [37-39]. Additionally, previous studies using the technology acceptance model also showed that the perceived usefulness of using apps is a positive predictor of the intention to use the apps [40-42]. In other words, believing that using cognitive training apps can improve cognitive function is an important factor in predicting the intention to use the apps. Moreover, this belief is also critical for the continued use of health apps [43,44].

Dementia knowledge was linked to the actual use of cognitive training apps in this study, which is consistent with previous reports showing that dementia knowledge is positively associated with preventive behavior for dementia [45-47]. A systematic review on dementia knowledge points out that most people think cognitive activities that exercise the brain are more effective in preventing dementia rather than medications, exercise, or dietary modifications [48], which may explain the link between dementia knowledge and the use of cognitive training apps.

Meanwhile, the average dementia knowledge score in this study was 9.05 points out of 15. Recent studies on middle-aged adults (40 to 75 years of age) showed that more than half of the respondents had insufficient dementia knowledge, 59.4% wanted information for cognitive health, and 70% had positive feelings for eHealth use to improve cognitive health [14,15]. As both the need and demand for dementia-related education is high, mobile apps can serve as a useful tool to deliver dementia knowledge and provide cognitive training programs in the middle-aged compared to the elderly, in terms of technology friendliness.

Our results showed that women were more likely to use cognitive training apps than men. This is consistent with a previous study, which showed women participate in activities that help improve cognitive health more frequently than men [49]. This may be linked to the higher prevalence of dementia among women compared with men [50], and the higher level of experience with dementia patients in females [1], although further studies are required to elucidate the exact relationships between these factors.

Finally, individuals with a family history of dementia were more likely to use cognitive training apps in this study. Previous studies show that people with a family history of dementia think that they have a higher risk of developing dementia, and as having a family history of AD has consistently emerged as a key predictor of dementia worry [51], they may undergo activities for dementia prevention more actively. However, our results showed that perceived susceptibility did not predict the use of cognitive training apps, similar to the findings of a previous report that showed self-perceived risk itself did not predict preventive behavior [52]. Other studies also show that people with family history of dementia are less likely to believe that dementia is preventable, and that they have lower self-efficacy for dementia prevention [53]. Therefore, it is important to understand the characteristics of this population and perform suitable interventions, which can lead to a positive attitude for dementia prevention.

### Limitations

The questionnaire data was collected on an online basis; therefore, individuals could have shown higher digital literacy compared with the normal population.

### Conclusions

This study explores the influencing factors on the experience of cognitive training apps using an extended HBM model, which may serve as a theoretical basis for the development of intervention strategies to increase the use of cognitive training apps for dementia prevention.

## Appendix

Multimedia Appendix 1 Full questionnaire (in Korean).

Multimedia Appendix 2 Full questionnaire (in English).

## Abbreviations

AD: Alzheimer disease
ANOVA: analysis of variance
HBM: health belief model
OR: odds ratio
