# Supplementary material for: Factors Associated With the Experience of Cognitive Training Apps for the Prevention of Dementia: Cross-sectional Study Using an Extended Health Belief Model
Source: J Med Internet Res. 2022 Jan 14;24(1):e31664. doi: 10.2196/31664 (PMC8800093; doi:10.2196/31664)
Supplement: Multimedia Appendix 1 [file jmir_v24i1e31664_app1.docx]

Multimedia Appendix 1. Full Questionnaire (in Korean)

**일반적 특성 - 9문항**

1. 출생년도 ________ 년

2. 성별 ① 남 ② 여

3. 귀하의 교육수준은 어떻게 되십니까?

① 무학 ② 초등학교 졸업 ③ 중학교 졸업 ④ 고등학교 졸업⑤ 2, 3년제 대학교 졸업 ⑥ 4년제 대학교 졸업 ⑦ 대학원 이상

4. 귀하의 결혼상태는 어떻게 되십니까?

① 미혼 ② 기혼(결혼) ③ 사별 ④ 별거/이혼 ⑤ 기타

5. 귀하의 동거상태는 어떻게 되십니까? 함께 거주중인 사람들을 모두 선택해주시기 바랍니다. (중복선택)

① 독거 ② 배우자 ③ 부모 ④ 자녀

⑤ 손자녀 ⑥ 기타

6. 귀하는 현재 앓고 있는 만성질환이 있습니까? (중복선택)

① 없음 ② 고혈압 ③ 당뇨병 ④ 고지혈증

⑤ 빈혈 ⑥ 만성콩팥병 ⑦ 만성간염 ⑧ 기타 ( )

7. 귀하의 가족(부모나 형제) 중 치매이신 분이 계십니까? 사망자를 포함하여 응답해주시기 바랍니다.

① 있음 ② 없음 ③ 잘 모르겠다

8. 귀하는 최근 한 달간 인터넷을 며칠이나 이용하셨습니까? (PC, 일반 휴대폰, 스마트폰, 스마트패드 등 모두 포함) ______ 일

9. 귀하는 스마트폰에서 인지기능 강화 앱을 사용해본 적이 있으십니까? (예시: 기억력게임, 두뇌운동 한글퀴즈, 성경카드메모리, 엔브레인 등)

① 있음 ② 없음

**치매지식 - 15문항**

치매에 대한 아래와 같은 생각은 맞다고 생각하십니까? 혹은 틀리다고 생각하십니까? 귀하의 의견을 말씀해 주십시오.

|  | 그렇다 | 아니다 | 잘 모르겠다 |
| --- | --- | --- | --- |
| 1) 나이가 들면 누구나 치매에 걸린다. | ① | ② | ③ |
| 2) 치매는 뇌의 병이다. | ① | ② | ③ |
| 3) 남자보다 여자가 치매에 더 잘 걸린다. | ① | ② | ③ |
| 4) 뇌졸중(중풍) 때문에 치매가 생길 수 있다. | ① | ② | ③ |
| 5) 술을 많이 마시면 치매에 걸리기 쉽다. | ① | ② | ③ |
| 6) 노인은 100명 중 한 명 꼴로 치매에 걸린다. | ① | ② | ③ |
| 7) 부모가 치매이면 자식도 치매에 걸리게 된다. | ① | ② | ③ |
| 8) 옛날 일을 잘 기억하면 치매가 아니다. | ① | ② | ③ |
| 9) 치매에 걸리면 성격이 변할 수 있다. | ① | ② | ③ |
| 10) 치매에 우울증이 잘 동반된다. | ① | ② | ③ |
| 11) 치매는 혈액 검사로 진단한다. | ① | ② | ③ |
| 12) 치매는 치료가 불가능하다. | ① | ② | ③ |
| 13) 일찍 치료를 시작하면 진행을 늦출 수 있다. | ① | ② | ③ |
| 14) 규칙적인 운동이 치매 예방에 도움이 된다. | ① | ② | ③ |
| 15) 치매 노인은 모두 장애인으로 등록할 수 있다. | ① | ② | ③ |

치매에 대한 개인적인 인식을 묻는 질문입니다. 귀하의 의견과 일치하는 곳을 선택해주시기 바랍니다.

**지각된 취약성 – 4문항**

|  | 전혀 그렇지 않다 | 그렇지 않다 | 잘 모르겠다 | 그렇다 | 매우 그렇다 |
| --- | --- | --- | --- | --- | --- |
| 1. 나는 같은 나이의 다른 사람에 비해 치매에 걸릴 가능성이 높다. | ① | ② | ③ | ④ | ⑤ |
| 2. 나는 나이가 들수록 치매에 걸릴 가능성이 높다. | ① | ② | ③ | ④ | ⑤ |
| 3. 가족 중에 치매에 걸린 사람이 있다면 나도 치매에 걸릴 것이다. | ① | ② | ③ | ④ | ⑤ |
| 4. 나는 치매에 걸릴 확률이 높다고 느낀다. | ① | ② | ③ | ④ | ⑤ |

**지각된 심각성 - 4문항**

|  | 전혀 그렇지 않다 | 그렇지 않다 | 잘 모르겠다 | 그렇다 | 매우 그렇다 |
| --- | --- | --- | --- | --- | --- |
| 1. 치매는 질병 중에서도 끔찍한 편에 속한다. | ① | ② | ③ | ④ | ⑤ |
| 2. 치매에 걸리면 목숨을 잃을 수도 있다. | ① | ② | ③ | ④ | ⑤ |
| 3. 나는 치매에 걸리는 것보다 갑작스러운 사고(예: 자동차 사고)로 죽는 것이 나을 것 같다. | ① | ② | ③ | ④ | ⑤ |
| 4. 나는 치매에 걸리는 것보다 다른 만성질환에 걸리는 것이 나을 것 같다. | ① | ② | ③ | ④ | ⑤ |

인지기능 강화 앱에 대한 설명입니다. 아래 설명과 이미지를 자세히 읽어주신 후, 제시되는 문항에 응답해주시기 바랍니다.

**인지기능 강화 앱이란 게임, 퍼즐, 퀴즈, 문제풀이 등의 다양한 형식을 통해 기억력, 주의력, 언어능력, 공간지각능력 등의 인지기능을 자극하는 프로그램을 제공하는 모바일 앱
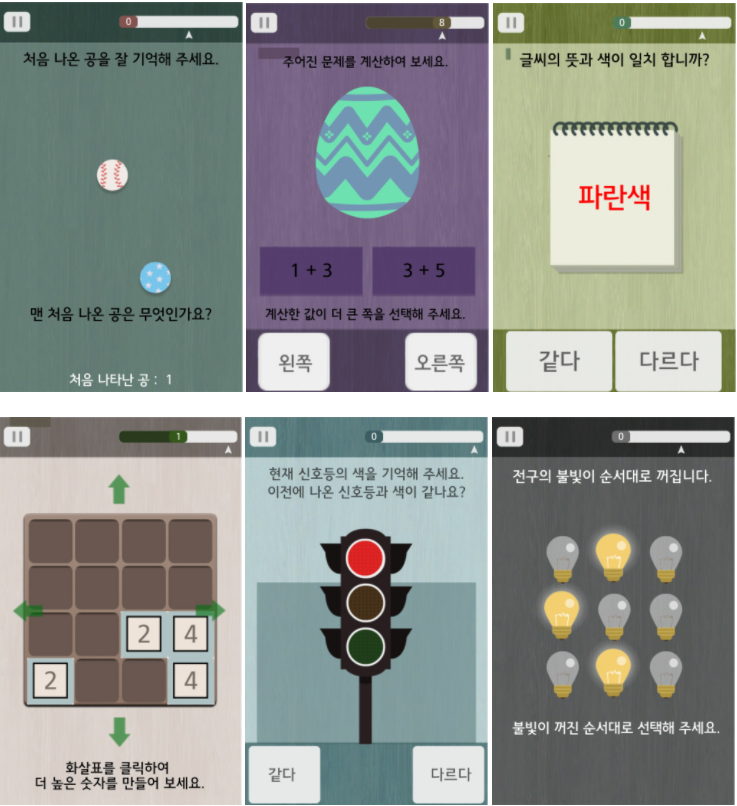
을 말한다.

[출처: 엔브레인]

인지기능 강화 앱 사용에 대한 개인적인 인식을 묻는 질문입니다. 귀하의 의견과 일치하는 곳을 선택해주시기 바랍니다.

**지각된 이익 - 4문항**

|  | 전혀 그렇지 않다 | 그렇지 않다 | 잘 모르겠다 | 그렇다 | 매우 그렇다 |
| --- | --- | --- | --- | --- | --- |
| 1. 인지기능 강화 앱을 통해 인지기능을 향상시킬 수 있다고 생각한다. | ① | ② | ③ | ④ | ⑤ |
| 2. 인지기능 강화 앱을 통해 인지기능을 더 효율적으로 향상시킬 수 있다고 생각한다. | ① | ② | ③ | ④ | ⑤ |
| 3. 인지기능 강화 앱을 통해 인지기능을 더 손쉽게 향상시킬 수 있다고 생각한다. | ① | ② | ③ | ④ | ⑤ |
| 4. 인지기능 강화 앱은 나에게 유용할 것이라고 생각한다. | ① | ② | ③ | ④ | ⑤ |

**지각된 장애 – 5문항**

|  | 전혀 그렇지 않다 | 그렇지 않다 | 잘 모르겠다 | 그렇다 | 매우 그렇다 |
| --- | --- | --- | --- | --- | --- |
| 1. 나는 인지기능 강화 앱 사용이 복잡하고 어렵다고 생각한다. | ① | ② | ③ | ④ | ⑤ |
| 2. 나는 인지기능 강화 앱 사용이 불편하다고 생각한다. | ① | ② | ③ | ④ | ⑤ |
| 3. 나는 인지기능 강화 앱이 제공하는 기능이 부족하다고 생각한다. | ① | ② | ③ | ④ | ⑤ |
| 4. 나는 인지기능 강화 앱을 이용할 때 시간이 너무 많이 걸린다고 생각한다. | ① | ② | ③ | ④ | ⑤ |
| 5. 나는 인지기능 강화 앱을 이용할 때 비용이 너무 많이 든다고 생각한다. | ① | ② | ③ | ④ | ⑤ |
