# Supplementary material for: Factors Associated With the Experience of Cognitive Training Apps for the Prevention of Dementia: Cross-sectional Study Using an Extended Health Belief Model
Source: J Med Internet Res. 2022 Jan 14;24(1):e31664. doi: 10.2196/31664 (PMC8800093; doi:10.2196/31664)
Supplement: Multimedia Appendix 2 [file jmir_v24i1e31664_app2.docx]

Multimedia Appendix 2. Full Questionnaire (in English)

**General Characteristics – 9 items**

1. Year of Birth __________

2. Gender ① Male ② Female

3. Education ① None ② Elementary school ③ Middle school

④ High school ⑤ College, 2~3 year course ⑥ College, 4 year course

⑦ Graduate school

4. Marital status ① Single ② Married ③ Widowed ④ Divorced ⑤ Other

5. With whom are you currently living with? (multiple choice question)

① Living alone ② Spouse ③ Parent

④ Children ⑤ Grandchildren ⑥ Other

6. Are you currently suffering from chronic diseases? (multiple choice question).

① None ② Hypertension ③ Diabetes

④ Hyperlipidemia ⑤ Anemia ⑥ Chronic kidney disease

⑦ Chronic hepatitis ⑧ Other ( )

7. Is there a dementia patient in your family (parents or siblings, including deceased)?

① Yes ② No ③ Don’t know

8. How many days have you used the Internet in the past month? (includes use with PC, cellphone, smartphone, smartpad, etc.) ________ days

9. Have you ever used cognitive training apps on your smartphone? (ex. Memory Games, Brain exercise Korean Quiz, Bible Card Memory, enBrain, etc)

① Yes ② No

**Dementia Knowledge (15 items)**

|  | Yes | No | Don’t know |
| --- | --- | --- | --- |
| 1. Everyone gets dementia as they age. | ① | ② | ③ |
| 2. Dementia is a disease of the brain. | ① | ② | ③ |
| 3. Women get dementia more frequently than men. | ① | ② | ③ |
| 4. Stroke can cause dementia. | ① | ② | ③ |
| 5. Heavy drinking increases the chance of getting dementia. | ① | ② | ③ |
| 6. One out of 100 older adults get dementia. | ① | ② | ③ |
| 7. If a parent has dementia, their children will also get dementia. | ① | ② | ③ |
| 8. If someone’s memories of old events are intact, he/she does not have dementia. | ① | ② | ③ |
| 9. Dementia can cause personality changes. | ① | ② | ③ |
| 10. Dementia is frequently accompanied by depression. | ① | ② | ③ |
| 11. Dementia is diagnosed by blood tests. | ① | ② | ③ |
| 12. Dementia is not treatable. | ① | ② | ③ |
| 13. Early treatment can delay the progression of dementia. | ① | ② | ③ |
| 14. Regular exercise can help prevent dementia. | ① | ② | ③ |
| 15. Any older adult with dementia can register as a disabled person. | ① | ② | ③ |

**Perceived Susceptibility of dementia (4 items)**

|  | Strongly  Disagree | Disagree | Neutral | Agree | Strongly  Agree |
| --- | --- | --- | --- | --- | --- |
| 1. Compared to other people of my age, I have a pretty good chance of getting dementia. | ① | ② | ③ | ④ | ⑤ |
| 2. As I age, I am more likely to get dementia. | ① | ② | ③ | ④ | ⑤ |
| 3. If a family member has dementia, then I will also get dementia. | ① | ② | ③ | ④ | ⑤ |
| 4. I feel that I have a high chance of getting dementia. | ① | ② | ③ | ④ | ⑤ |

**Perceived Severity of dementia (4 items)**

|  | Strongly  Disagree | Disagree | Neutral | Agree | Strongly  Agree |
| --- | --- | --- | --- | --- | --- |
| 1. Dementia is a horrible disease. | ① | ② | ③ | ④ | ⑤ |
| 2. If I get dementia, I can die from it. | ① | ② | ③ | ④ | ⑤ |
| 3. I would rather die from a sudden accident (ex. Car crash) than get dementia. | ① | ② | ③ | ④ | ⑤ |
| 4. I would rather get a chronic disease than get dementia. | ① | ② | ③ | ④ | ⑤ |

Cognitive training apps are mobile applications that utilize various methods such as games, puzzles, quizzes, or problem solving to stimulate cognitive functions including memory,
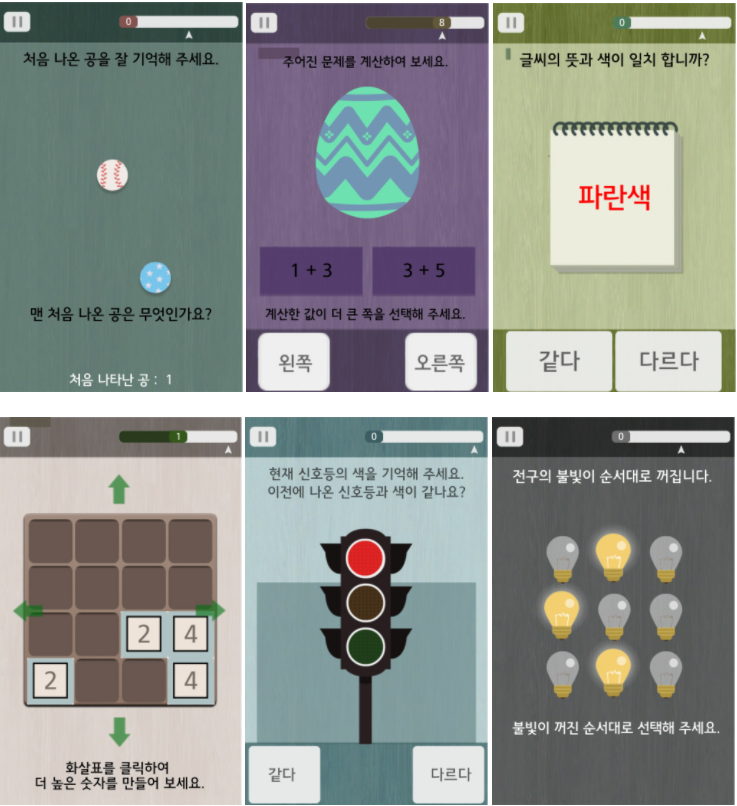
attention, language skills, and spatial perception.

[Reference: enBrain]

**Perceived Benefit of using cognitive training apps (4 items)**

|  | Strongly  Disagree | Disagree | Neutral | Agree | Strongly  Agree |
| --- | --- | --- | --- | --- | --- |
| 1. Using cognitive training apps can improve my cognitive functions. | ① | ② | ③ | ④ | ⑤ |
| 2. Using cognitive training apps can improve my cognitive functions more efficiently. | ① | ② | ③ | ④ | ⑤ |
| 3. Using cognitive training apps can improve my cognitive functions more easily. | ① | ② | ③ | ④ | ⑤ |
| 4. I find cognitive training apps to be useful. | ① | ② | ③ | ④ | ⑤ |

**Perceived Barrier of using cognitive training apps (5 items)**

|  | Strongly  Disagree | Disagree | Neutral | Agree | Strongly  Agree |
| --- | --- | --- | --- | --- | --- |
| 1. Using cognitive training apps is complicated and difficult. | ① | ② | ③ | ④ | ⑤ |
| 2. Using cognitive training apps is uncomfortable. | ① | ② | ③ | ④ | ⑤ |
| 3. Cognitive training apps provide only limited functions. | ① | ② | ③ | ④ | ⑤ |
| 4. Using cognitive training apps takes too much time. | ① | ② | ③ | ④ | ⑤ |
| 5. Using cognitive training apps is too expensive. | ① | ② | ③ | ④ | ⑤ |
